# Response: Commentary: Efficacy of Follicle-Stimulating Hormone (FSH) Alone, FSH + Luteinizing Hormone, Human Menopausal Gonadotropin or FSH + Human Chorionic Gonadotropin on Assisted Reproductive Technology Outcomes in the “Personalized” Medicine Era: A Meta-analysis

**DOI:** 10.3389/fendo.2018.00113

**Published:** 2018-03-23

**Authors:** Daniele Santi, Livio Casarini, Carlo Alviggi, Manuela Simoni

**Affiliations:** ^1^Unit of Endocrinology, Department of Biomedical, Metabolic and Neural Sciences, University of Modena and Reggio Emilia, Modena, Italy; ^2^Unit of Endocrinology, Department of Medicine, Endocrinology, Metabolism and Geriatrics, Azienda Ospedaliero-Universitaria of Modena, Modena, Italy; ^3^Department of Neuroscience, Reproductive Science and Odontostomatology, University of Naples Federico II, Napoli, Italy

**Keywords:** follicle-stimulating hormone, luteinizing hormone, human chorionic gonadotropin, human menopausal gonadotropin, pregnancy rate, assisted reproductive technology, controlled ovarian stimulation

We appreciated the constructive comments by Professor Younis (1) to our article (2). He gave us the opportunity to further discuss issues raising by the lack of clear evidence in the setting of assisted reproduction techniques (ART).

Although 65 meta-analyses were published so far, our study represents the first attempt to consider all gonadotropin combinations currently used in clinical practice, mainly focusing on specific effects linked to luteinizing hormone (LH) or human choriogonadotropin (hCG) supplementation. The evaluation of how different therapeutic approaches impact on peculiar clinical settings, such as polycystic ovarian syndrome or hypo-responder women, was beyond the aims of the study. It would have determined a complicated stratification process, where many grouping variables would have been evaluated in too many undersized subgroups. Moreover, we agree with Professor Younis about the relevance of genetic characteristics. The polygenic nature of ovarian response implies that the ideal approach to controlled ovarian stimulation (COS) should be patient-specific and determined by genetic screening.

Recently, the Cochrane Collaboration evaluated the effect of LH addition during COS on live birth rate, placing no limit of age or clinical setting (3). In spite of the strict selection of randomized trials, the risk of selection biases remained relatively high. This was due to potential errors introduced during the selection of participants, since the study groups may not be effectively representative of the population. Moreover, the method used for randomization is not clearly described in 62.1% of all controlled clinical trials published in the setting of ART (4). We decided not to consider randomization for selection, aiming at (I) increasing the number of potential studies to be enrolled and (II) reducing possible publication biases. The exclusion of *non*-randomized trials from our meta-analysis would had significantly reduced the number of studies and of patients evaluated, limiting the final statistical significance of the result. Anyway, and as recommended by the PRISMA statement, we set strict inclusion and exclusion criteria before literature search.

We agree that women’s age is an important parameter driving clinicians’ decision toward the best COS scheme. In our meta-analysis, only the most important results were reported in the main paper, such as the type of gonadotropin-releasing hormone (GnRH) analog used. However, we considered age, and these data are reported in the supplementary materials of the original manuscript. In this case, a reasonable cutoff age of 35 years was chosen, which is an empirical matter in clinical practice as well. Asymmetric subdivision of studies was obtained, with only 17 out of 70 studies (24.3%) enrolling women with a mean age of >35 years. Although this cutoff could have application in clinical practice, it is not methodologically acceptable in a meta-analysis. Interestingly, we confirmed the results obtained in the overall analysis, when LH + FSH was compared to FSH alone, by considering the median value of women’s age as a cutoff (32.5 years). Results obtained comparing hMG vs. FSH were confirmed too in the group of women younger than 32.5 years. Taken together, these data suggest the importance of age for COS protocol personalization, but the empirical nature of the selected cutoffs is methodologically arbitrary.

In ART, women are usually characterized as poor, normo-, and high-responders (5). While this classification is useful for clinicians to choose the proper COS protocol, it could not be used in our research. Although several clinical trials are focused on poor-responder women, not all studies reported the classification as an inclusion criterion. Moreover, two meta-analyses comparing LH + FSH vs. FSH alone in poor responders were previously published showing controversial results (6, 7). A debate about the definition of poor, normo-, and high-responders is still open, and different criteria were proposed (8, 9). Furthermore, concepts of suboptimal and hyporesponse are also emerging (10–12), but no trials in women accordingly selected have been published so far. Finally, consensus definition of poor responders is available but it is matter of debate and apparently not able to select homogenous study populations. Taken together, these observations confirm the weakness of this parameter both in clinical practice (13) and research setting.

It is common opinion that the type of GnRH analog used provides different residual LH activity, relying on the concept that <1% of LH receptors (LHCGR) occupied by the ligand are enough to elicit steroid synthesis (14). This is the concept known as “spare receptors” emerging from previous studies (15), often improperly handled by gynecologists to interpret and transpose the messages provided by *in vitro* studies to the clinics. The concept originates from the historical investigations of Dufau and Catt (15), who evaluated hCG-mediated cAMP increase and testosterone synthesis in rat Leydig cells, rather than human ovarian and endometrial cells treated by LH. The findings of such studies explained pharmacological mechanisms assessed exclusively in a specific context *in vitro* which cannot be used to explain clinical observations. In light of recent studies demonstrating fivefold lower LH- than hCG-mediated cAMP increase (16–18), as well as the inability of Leydig cells expressing rodent receptors to discriminate qualitatively intracellular signals mediated by the two human ligands (18), we should seriously reconsider if residual LH (putative) activity is mediated by LHCGR in granulosa cells. LH is not as potent as hCG in inducing steroidogenic signals. On the other hand, LH displays preferential proliferative signals in human granulosa cells (19), not appraisable in rodents’ Leydig cells, unable to discriminate qualitatively between LH- and hCG-specific signals. Rather, residual LH activity should be due to FSH receptor-LHCGR heterodimers activated by FSH and mediating LH-like stimuli. This is also suggested by animal models: while FSH alone triggers follicle maturation and ovulation in hypophysectomized mice, it fails to do the same in LH receptor-knockout mice (20). Anyway, since the residual LH activity is not routinely evaluated in clinical trials, this could not be considered in our meta-analysis.

Prof. Younis’ comments shift the interpretation of our findings toward a clinical point of view, focusing on the detection of parameters useful to choose the best COS protocol, as a crucial issue for personalized medicine. However, meta-analyses fall short to provide guidelines or recommendations for clinical practice, since relying on data already published, which could not be directly exported to the general population. Starting from different activities demonstrated *in vitro* (16), our study aimed at evaluating the different action of gonadotropins *in vivo*, considering the COS phase as an experimental model in which to test these molecules. Therefore, our meta-analysis is a starting point to design clinical trials aimed at evaluating the best gonadotropins combination for ART.

## Author Contributions

DS, LC, CA, and MS contributed to the manuscript draft.

## Conflict of Interest Statement

The authors declare that the research was conducted in the absence of any commercial or financial relationships that could be construed as a potential conflict of interest.
